# Prolonged Heat Acclimation and Aerobic Performance in Endurance Trained Athletes

**DOI:** 10.3389/fphys.2019.01372

**Published:** 2019-11-01

**Authors:** C. Jacob Mikkelsen, Nicklas Junge, Jacob F. Piil, Nathan B. Morris, Laura Oberholzer, Christoph Siebenmann, Carsten Lundby, Lars Nybo

**Affiliations:** ^1^Department of Nutrition, Exercise and Sports, University of Copenhagen, Copenhagen, Denmark; ^2^Centre for Physical Activity Research, Copenhagen University Hospital, Copenhagen, Denmark; ^3^Institute of Mountain Emergency Medicine, EURAC Research, Bolzano, Italy; ^4^Innland Norway University of Applied Sciences, Lillehammer, Norway

**Keywords:** cycling time trial, maximal oxygen uptake, exercise, peak power output, cycling efficiency

## Abstract

Heat acclimation (HA) involves physiological adaptations that directly promote exercise performance in hot environments. However, for endurance-athletes it is unclear if adaptations also improve aerobic capacity and performance in cool conditions, partly because previous randomized controlled trial (RCT) studies have been restricted to short intervention periods. Prolonged HA was therefore deployed in the present RCT study including 21 cyclists [38 ± 2 years, 184 ± 1 cm, 80.4 ± 1.7 kg, and maximal oxygen uptake (VO_2max_) of 58.1 ± 1.2 mL/min/kg; mean ± SE] allocated to either 5½ weeks of training in the heat [HEAT (*n* = 12)] or cool control [CON (*n* = 9)]. Training registration, familiarization to test procedures, determination of VO_2max_, blood volume and 15 km time trial (TT) performance were assessed in cool conditions (14°C) during a 2-week lead-in period, as well as immediately pre and post the intervention. Participants were instructed to maintain total training volume and complete habitual high intensity intervals in normal settings; but HEAT substituted part of cool training with 28 ± 2 sessions in the heat (1 h at 60% VO_2max_ in 40°C; eliciting core temperatures above 39°C in all sessions), while CON completed all training in cool conditions. Acclimation for HEAT was verified by lower sweat sodium [Na^+^], reduced steady-state heart rate and improved submaximal exercise endurance in the heat. However, when tested in cool conditions both peak power output and VO_2max_ remained unchanged for HEAT (pre 60.0 ± 1.5 vs. 59.8 ± 1.3 mL O_2_/min/kg). TT performance tested in 14°C was improved for HEAT and average power output increased from 298 ± 6 to 315 ± 6 W (*P* < 0.05), but a similar improvement was observed for CON (from 294 ± 11 to 311 ± 10 W). Based on the present findings, we conclude that training in the heat was not superior compared to normal (control) training for improving aerobic power or TT performance in cool conditions.

## Introduction

It is well documented that natural heat acclimatization as well as laboratory-based heat acclimation (HA) improve exercise performance in hot environments (see Daanen et al., 2018 for review and Lorenzo et al., 2010; Karlsen et al., 2015a, b; Racinais et al., 2015 for specific studies). In contrast, if HA also leads to physiological adaptations that will improve exercise performance in cool conditions remains controversial (Corbett et al., 2014; Minson and Cotter, 2016; Nybo and Lundby, 2016). On one hand, Scoon et al. (2007), Karlsen et al. (2015b), and Keiser et al. (2015) report no effect of HA (or similar performance effects as reported for a matched control group training in cool settings) on exercise endurance performance in cool conditions. On the other hand, a few randomized controlled trials (RCTs) studies have reported beneficial effects (Lorenzo et al., 2010; McCleave et al., 2017; Rendell et al., 2017) on VO_2max_, time trial (TT), and lactate threshold. Furthermore, studies that did not include a control group in the study design also report that training in the heat may benefit aerobic performance in cool setting (Hue et al., 2007; Buchheit et al., 2011, 2013; Racinais et al., 2014; Neal et al., 2016a, b). The difference in findings from the above studies may, to some extent, relate to the heterogeneity of the studies, as they differ in the participants’ training status, the conditions undertaken by the control group and, in particular, the duration of the intervention period. To conclude if HA may translate into improved aerobic performance in cool conditions, it is not sufficient to merely demonstrate improved performance in untrained or recreationally active adults, as improvements in performance could relate to a standard training effect rather than environmental stress. Also, for athletes, the improvement accomplished by training in the heat would need to be superior compared to control training that includes high-intensity intervals (Levine and Stray-Gundersen, 1997).

The proposed ergogenic effect of HA for subsequent performances in cool conditions has been attributed to a combination of hematological, cardiovascular, and skeletal muscle adaptations (Corbett et al., 2014). One mechanism of particular interest has been the expansion of plasma volume (and general increase of extra-cellular volume; see Patterson et al., 2004) that could translate into increased hemoglobin mass (Hb_mass_) and higher capacity for systemic oxygen delivery (Scoon et al., 2007); although the erythropoietic effect was not observed in the study by Patterson et al. (2004). Diverse effects on Hb_mass_ as well as plasma and blood volume across available HA studies may relate to relative short intervention periods deployed in previous studies allowing limited time for erythropoiesis to occur. In that context, significant effects of environmental interventions, e.g., altitude exposure is typically considered to require several weeks depending on the strength of the stimuli (Rasmussen et al., 2013; Siebenmann et al., 2015). Therefore, to determine whether HA positively enhances erythropoiesis in trained subjects, HA studies with sufficient duration are warranted.

In addition to central hemodynamic responses, peripheral adaptations of relevance for performance have been proposed to be enhanced by heat training (Coyle, 1999; Bassett and Howley, 2000). For example, improved gross efficiency (GE) following heat training has been observed in some studies (Shvartz et al., 1977; Sawka et al., 1983) but not in others (Karlsen et al., 2015b; Rendell et al., 2017). While enhanced exercise efficiency has been proposed to involve changes in skeletal muscle recruitment patterns in response to heating (Shvartz et al., 1977; Sawka et al., 1983; Corbett et al., 2014), the improved efficiency could merely relate to acquaintance with the experimental testing as the studies reporting this effect have not controlled for familiarization effects.

Therefore, the present study was conducted to evaluate the efficacy of prolonged HA (induced by training in the heat) compared to continued normal training (in settings with low thermal stress) with focus on the potential for improving exercise performance and aerobic capacity in cool conditions. Specifically, a long-term (5½ weeks) laboratory-based heat-training period was employed to ensure adequate time for any potential erythropoietic effect to occur. We included a group of endurance trained male cyclists that, following familiarization and a controlled lead-in phase, were randomly allocated to a heat training (HEAT) or control (CON) group. It was hypothesized that long-term heat training would improve aerobic capacity, peak power output and prolonged exercise performance, as determined by a 15-km time-trial (TT). We evaluated if potential performance effects involved improved exercise efficiency, thermoregulatory factors related to sudomotor adaptations and hematological adaptations leading to plasma and blood volume expansion. The present paper is focused on the overall performance effects (TT, peak power, and aerobic capacity), while we refer to the accompanying publication by Oberholzer et al. (2019, submitted to the special issue) for details on the hematological adaptations and in-depth analyses of mechanisms involved.

## Materials and Methods

### Participants

Twenty-four well-trained, sub-elite male cyclists [38 ± 9 years, 184 ± 4 cm, 80.4 ± 8.0 kg, and maximal oxygen uptake (VO_2max_) of 58.1 ± 5.3 mL/min/kg; Mean ± SD] with at least 3 years of cycling experience were initially recruited (see Table 1 for group-specific overview of baseline descriptive data). Participants had conducted their usual off-season training (environmental temperatures <15°C) leading up to the study and were thus assumed to be only partly heat acclimated due to training status (Armstrong and Maresh, 1991). Following pre-intervention testing, participants were block-allocated into two performance-, VO_2max_-, and age-matched groups (*n* = 12) that subsequently were randomly designated as either the heat training (HEAT) or control (CON) group, however, due to personal reasons unrelated to the study, three subjects withdrew before commencement of the intervention, resulting in 12 and 9 participants in the HEAT and CON completing the study, respectively. Data from drop-outs were excluded from the analysis. Before providing their written consent to participate, subjects were informed of potential risks and discomforts associated with the experimental procedures. The study was conducted in accordance with the Helsinki declaration and approved by the ethics committee of the Capital Region of Denmark (protocol: H-17036662).

**TABLE 1:** Baseline descriptive data of participants and training characteristics before (during lead-in phase) and during the intervention for the heat training group (HEAT) and control group (CON).

	**Heat (*n* = 12)**	**Con (*n* = 9)**
Age (years)	39 ± 9	38 ± 9
Height (cm)	185 ± 3	183 ± 5
Body mass (kg)	80.2 ± 6.3	80.5 ± 9.5
Body fat percentage (%)	13.7 ± 3.9	14.7 ± 2.9
VO_2max_ (L/min)	4.8 ± 0.4	4.6 ± 0.4
VO_2max_/kg (mL/min/kg)	60.0 ± 5.1	57.9 ± 5.1
iPPO (W)	409 ± 20	408 ± 33
iPPO/kg (W/kg)	5.1 ± 0.5	5.1 ± 0.6
Training volume (min/week – lead in)	417 ± 105	499 ± 164
Training volume (min/week – during)	509 ± 173^∗^	576 ± 143^∗^
Intense training (min/week > 80% HR_max_)	102 ± 71	102 ± 55
Intense training during intervention (min/week)	157 ± 90^∗^	122 ± 57^∗^

*^∗^Denotes a main effect of time compared to lead in (*P* < 0.05). All other comparisons were not different (*P* > 0.05). Values are mean ± SD.*

### Study Overview

**FIGURE 1:**
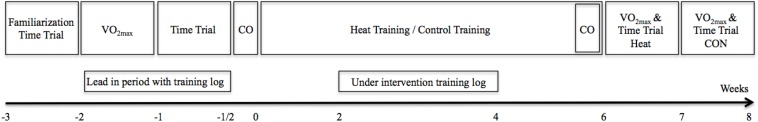
Study overview and time course. CO, carbon monoxide rebreathing procedure.

An overview of the study protocol is displayed in Figure 1. Upon enrolment into the study, participants first completed one familiarization session, completing a 30 min preload, followed by a 15 km TT. During the following 2 weeks, participants were monitored in a lead in phase, with registration of weekly training volume – both total and high intensity. On three subsequent sessions, baseline assessment of VO_2max_, cycling efficiency and TT performance, as well as hematological parameters (see accompanying paper by Oberholzer et al., 2019 for details) were conducted. Participants were then allocated to their respective groups and trained in a 5½ week period (see “Intervention Period” section for more detail). Following the training period, a post-test battery identical to the pre-intervention battery was conducted. All performance testing and training was conducted at the same time of day (within 2 h) using the participants’ personal bikes installed in a stationary Tacx-trainer device (Tacx Neo Smart T2800, Wassenaar, Netherlands) and associated software (Tacx Trainer software 4, Wassenaar, Netherlands). For each subject the same personal bike and Tacx-trainer were used during both pre- and post-intervention testing to circumvent any equipment differences. Pre-intervention performance testing was conducted during a 2-week period preceding the intervention (hematology within 2 days) and post-intervention testing within 6 days of the intervention’s conclusion. A recovery period lasting a minimum of 24 h separated all performance tests to preclude residual fatigue confounding the results. Subjects were instructed to abstain from performing any exhaustive exercise the day leading up to a performance test and to refrain from consumption of caffeine for 12 h and alcohol for 24 h prior to testing.

### Testing

#### Time Trial

Endurance performance was evaluated through the fastest possible completion of a simulated 15 km TT (mean slope of 0.1%, ∼600 m of uphill cycling) preceded by a 30-min preload at 60% of VO_2max_. The TT and preload were separated by 5 min of passive rest. Subjects had visual access to real-time information regarding heart rate (HR), distance completed/remaining, speed, cadence, and power output, but were blinded to elapsed time. The TT was conducted in temperate ambient conditions (14 ± 0.2°C, RH: 54 ± 3%; Galloway and Maughan, 1997) with airflow of ∼3 m/s directed toward the subjects’ frontal surface [WBGT of ∼10°C (Reed Heat Index checker 8778, Reed Instruments, United States)]. Provision of a maximal effort was facilitated by verbal encouragement throughout the test in addition to a prize being rewarded for the best performance.

#### VO_2max,_ Cycling Efficiency, Incremental Peak Power Output, and Anthropometry

Upon arrival to the laboratory and prior to performing any exercise, body mass and fat percentage were quantified on an electronic bio-impedance scale (InBody 270, InBody, Denmark). Cycling efficiency, VO_2max_, and incremental peak power output (iPPO) were assessed through completion of an incremental cycling test to volitional exhaustion. Following warm-up stages consisting of 5 min at 100 W and 5 min at 175 W (80 RPM), respectively, the work load was increased by 25 W/min, terminating when the subject was incapable of maintaining a pre-defined and self-selected cadence despite strong verbal encouragement. Breath by breath recordings of VO_2_ and VCO_2_ were obtained throughout the test [Jaeger Oxycon Pro, Viasys Healthcare, Germany (calibrated for room humidity, flow, and O_2_/CO_2_ concentration prior to each test)] and subsequently interpolated to 5 s mean values. Values ≥4 standard deviations from the local mean were discarded. A plateau in VO_2_ despite increased work load and/or attainment of a respiratory exchange ratio (RER) ≥1.15 served as test validation criteria. VO_2max_ was defined as the highest observed value over a 30-s period and iPPO as the last completed work stage (W) plus the fraction (s) of the last non-completed stage [iPPO = (Last completed work stage (W)) + (25 W/60 × *t*(*s*)].

### Intervention Period

Participants in the HEAT group underwent 60-min heat training sessions in a climatic chamber on 5 weekly occasions (28 ± 2 total sessions), while subjects in CON reported to the laboratory and trained once a week in cool conditions (∼15°C) to minimize group differences in the level of familiarization to stationary cycling and completed all other training and habitual intervals in cool settings. Both groups performed this part of the training at a constant intensity corresponding to 60% of VO_2max_ (204 ± 3 W). For HEAT, ambient temperature was set at 35°C the first week (3 days) and subsequently increased by one degree each week ending at 40°C (RH: 30 ± 2%), in order to accommodate for the relative decrease in intensity as HA was induced (Daanen et al., 2018). Rectal core temperature (T_core_) was elevated to ≥38.5°C after 35 ± 8 min of training and end T_core_ was 39.6 ± 0.4°C in all training sessions. Subjects were encouraged to undertake each training session without airflow for as long as subjectively tolerated but were provided with individually adjusted airflow when requested (ventilation with a floor fan of ∼1–3 m/s) to facilitate evaporation and provide some perceptual benefit to ensure that the exercise component could be completed. HR, T_core_, and sweat Na^+^ were quantified during the first and last weekly training session. Warm water was ingested *ad libitum* during training to avoid fluid consumption acting as a heat sink. Body mass was measured before and after each training session and subjects were instructed to replenish 150% of lost fluid during the following hours to re-establish euhydration. During the entirety of the intervention period, outside environmental temperature did not exceed 15°C.

### Confirmation of Acclimation Status Testing

To confirm acclimation status, a sub group of six participants from HEAT underwent a heat tolerance test (HTT) on day 1, 14, and 28. In order to avoid any partial HA, none of the participants from the CON group completed HTT testing. The HTT was conducted as a time to exhaustion (TTE) test under standardized 40°C at 60% VO_2max_, with no access to fan or other cooling (see “Measurement” section for details). Additionally, all participants in HEAT, were monitored for HR, T_core_, and changes in sweat Na^+^ concentration, in the beginning and end of all weeks of training.

### Measurements

#### Heart Rate and Core Temperature

Heart Rate was assessed using participants’ personal HR monitors (Garmin edge 500/520/820/1000/1030, Garmin Ltd., United States), was provided as a continuous feedback tool during TT, and was logged for acclimation status. T_core_ was recorded by a flexible rectal probe (Ellab, Denmark) self-inserted ∼10 cm beyond the anal sphincter. Both HR and T_core_ were measured during the first and the last HA training of all weeks of training as well during the HTT. Values were manually logged every 10 min and at TTE.

#### Sweat Rate

To calculate sweat rate (adjusted for fluid consumption) and to account for the effect of body mass during the TT, body mass (towel dried while wearing cycling shorts) was measured (InBody 270, InBody, Denmark) prior to the preload and following the TT. Sweat was obtained for Na^+^ content analysis (ABL 800 Flex, Radiometer, Denmark) by absorbent pads (Tegaderm +Pad, 3M, Denmark) placed on the upper back at the level of the scapulae, after thorough cleansing of the skin with demineralized water.

#### Training and Training Quantification

Training quantification was carried out during a 2-week lead in phase prior to the intervention and a 2-week period during the intervention, to quantify the impact of the intervention on participants’ habitual training procedures. Participants were instructed to fill out a training log containing information regarding total weekly training volume and total high-intensity training volume, the latter defined as training at HR above 80% of maximum (Karlsen et al., 2015b). Both groups were instructed to preserve their usual interval training routines alongside the intervention and to subtract the training associated with the intervention from their habitual training, to maintain total training volume as reported in their initial training log reported in the lead in phase.

#### Cycling Efficiency

Gross efficiency was calculated as the ratio of external mechanical work (W) to energy expenditure (EE). EE was calculated from steady state VO_2_ (confirmed in each participant by visual inspection of the VO_2_-time curve), RER and corresponding VO_2_ values obtained during the last 90 s of exercise at 100 (GE100) and 175 W (GE175), respectively, completed with fixed cadence of 80 RPM:

$$\text{GE=}\,\left(\frac{\frac{\text{watt}}{1000}*60}{\left(\left(\left(\frac{1-\mathrm{RER}}{0,3}\right)*19,3\right)+\left(\left(\frac{\mathrm{RER}-0,7}{0,3}\right)*21,1\right)\right)*\left(\frac{{\text{VO}}_{2}\,\frac{\text{ml}}{\text{min}}}{1000}\right)}\right)$$

### Statistical Analyses

All data are expressed as mean values with standard error unless otherwise stated. Pre-intervention group characteristics, performance results, and training logs were assessed using a student’s independent *T*-test to confirm homogeneity between groups. The changes pre- to post-intervention values between the heat training and control group for TT power output and completion time, relative, and absolute VO_2max_, iPPO, GE100, GE175, training time, and training time above 80% HR max were assessed with a two-way mixed-measures ANOVA, with the repeated factor of time point (two levels: Pre and Post) and the independent factor of intervention (two levels: HEAT and CON). Adaptations (T_core_, HR, sweat rate, and sweat Na^+^) in the HEAT group during the intervention period were evaluated with a paired samples *T*-test. When applicable, *post hoc* testing was carried out using a Holm–Sidak test. The probability of making a Type 1 error in all tests was maintained at 5%. All statistical analyses were carried out using GraphPad Prism (version 7.0, GraphPad Software, La Jolla, CA, United States).

## Results

### Heat Training and Acclimation Effects

In the HEAT group T_core_ increased during each training session in the heat and was above 39°C at the end of all sessions but decreased from the initial to the last training session (39.9 ± 0.4 vs. 39.4 ± 0.3°C, *P* < 0.05; see the weekly progression in Table 2). Similarly, end-training HR declined during the first 2 weeks of training in the heat and was significantly lowered from the first to the last training session in the heat (169 ± 10.5 vs. 155 ± 17 BPM, *P* < 0.05). In addition, sweat Na^+^ was reduced by 46 ± 14%, from 91 ± 5 mmol/L during the first week to 69 ± 8 mmol/L (*P* < 0.01), in the third week of training in HEAT, but did not decrease thereafter (see Table 2). Sweat rate during the cool TT testing did not significantly change from Pre to Post in either group (HEAT: 0.83 ± 0.03 vs. 0.93 ± 0.06 L/h; CON: 0.78 ± 0.03 vs. 0.78 ± 0.07 L/h, *P* > 0.05). The sub group of six participants, who underwent the HTT at 40°C, extended their TTE by 25.6 min (Day 1: 38.7 ± 2.4 vs. day 28: 64.3 ± 2.9 min, *P* < 0.001), from the first to the last test.

**TABLE 2:** Sweat sodium concentration, end-training HR, and rectal temperatures (*n* = 12) during the intervention for the heat training group (HEAT).

	**Sweat [Na^+^] (mmol l^–1^)**	**End-exercise HR (bpm)**	**Start rectal temperature (°C)**	**End-training rectal temperature (°C)**
First heat session	93 ± 5	167 ± 5	37.7 ± 0.2	39.9 ± 0.1
End of week 1	74 ± 5^∗^	160 ± 4	37.2 ± 0.3^∗^	39.7 ± 0.1
End of week 2	71 ± 6^∗^	160 ± 3^∗^	37.2 ± 0.2^∗^	39.7 ± 0.1
End of week 3	69 ± 7^∗^	155 ± 4^∗^	–	39.4 ± 0.1^∗^
End of week 4	72 ± 7^∗^	156 ± 4^∗^	37.0 ± 0.3^∗^	39.3 ± 0.1^∗^
End of week 5	79 ± 6^∗^	156 ± 4^∗^	–	39.4 ± 0.1^∗^

*^∗^Denotes a significant reduction compared to first training session (*P* < 0.05). Values are mean ± SE.*

### Time Trial in Cool Conditions

**FIGURE 2:**
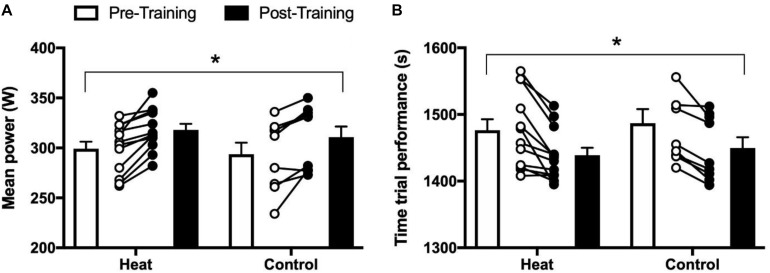
**(A)** Mean power output in watts and **(B)** time trial performance in seconds, during a 15 km time trial in cool/temperate conditions, for the intervention group (HEAT) and a control group. Open bars and circles illustrate average and individual measurements, respectively, before intervention, black bars and circles illustrate average and individual measurements, respectively, after intervention. ^∗^Denotes a significant main effect of time (*P* < 0.05). Values are shown as mean ± SE.

There was a main effect of time for average TT power output (+5.8 ± 0.9%, *P* < 0.001, see Figure 2), resulting from significant increases in both groups (HEAT: +6.0 ± 1.1%, *P* < 0.05; CON: +5.5 ± 1.6%, *P* < 0.05). Accordingly, TT performance time significantly decreased in both HEAT (by 37.4 ± 8.6 s, *P* < 0.05) and CON (37.1 ± 10.1 s, *P* < 0.05), with similar improvements across groups (i.e., there was no group × time interaction effect).

### VO_2max_, Incremental Peak Power Output, and Cycling Efficiency

**FIGURE 3:**
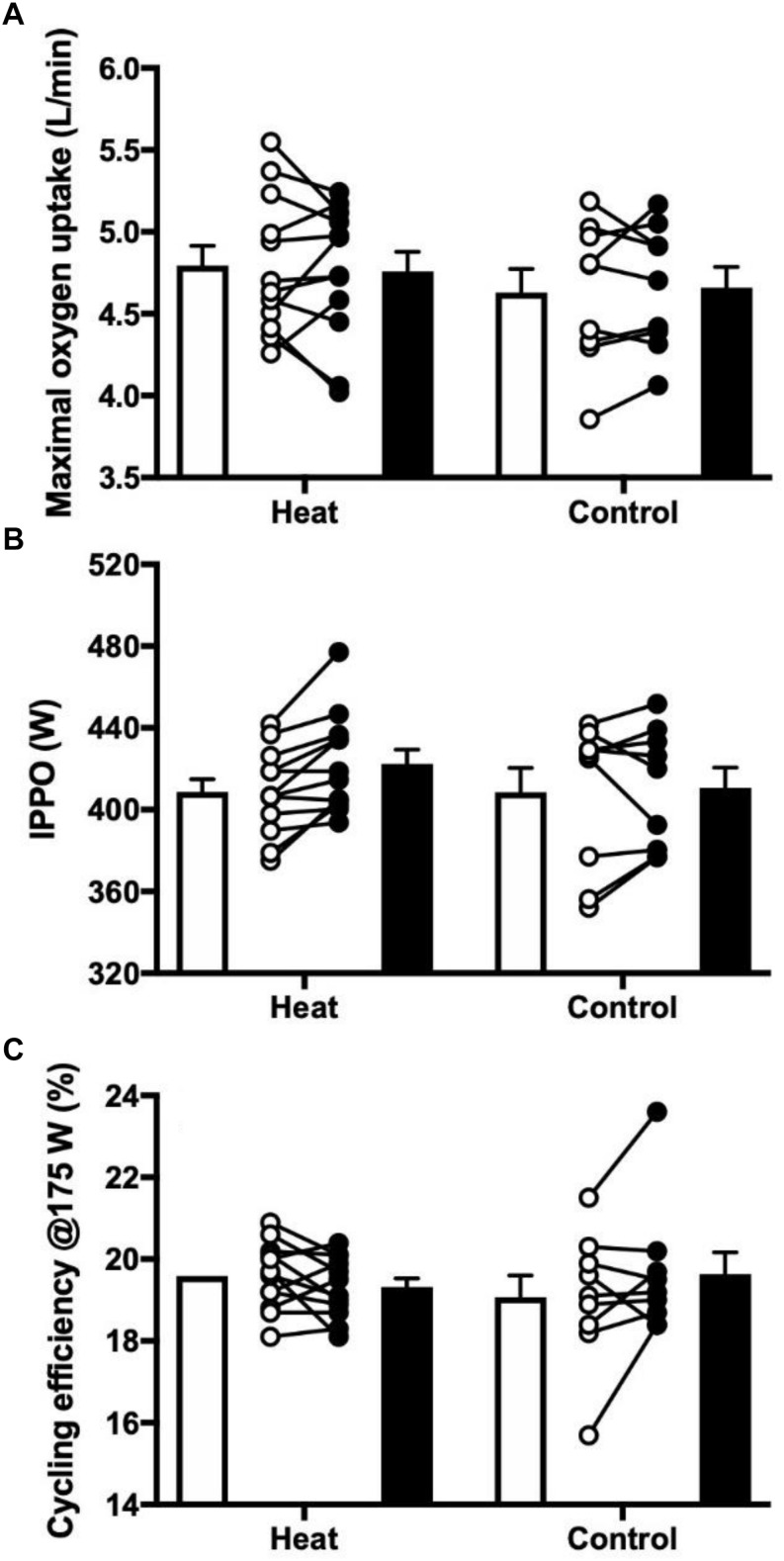
**(A)** Maximal oxygen uptake, **(B)** incremental peak power output in watts, and **(C)** cycling efficiency in percent at 175 W during an incremental test to exhaustion in cool/temperate conditions (∼13°C). Open bars and circles illustrate average and individual measurements, respectively, before intervention, black bars and circles illustrate average and individual measurements, respectively, after intervention. Values are shown as mean ± SE.

There was no change from pre to post in VO_2max_ in either group [HEAT: 4.8 ± 0.1 vs. 4.8 ± 0.1 L/min; CON: 4.6 ± 0.1 vs. 4.7 ± 0.1 L/min (*P* > 0.05, Figure 2)], and since there was no change in body weight from pre to post, relative VO_2max_ (mL O_2_/min/kg) also remained similar for both groups [HEAT: 60.0 ± 1.5 vs. 59.8 ± 1.2 mL/min/kg; CON 57.9 ± 1.7 vs. 59.4 ± 2 mL/min/kg (*P* > 0.05, see Figure 3)].

There was no main effect or significant effect of HA on iPPO [HEAT: 409 ± 6 vs. 422 ± 7 W; CON: 408 ± 11 vs. 411 ± 9 W (*P* > 0.05, Figure 3)], and cycling efficiency, evaluated as GE at 100 W (HEAT: 15.4 ± 0.3 vs. 15.2 ± 0.2%; CON: 14.8 ± 0.5 vs. 15.2 ± 0.4%) or 175 W (HEAT: 19.7 ± 0.3 vs. 19.4 ± 0.2%; CON: 19.3 ± 0.5 vs. 19.8 ± 0.5%), did not display any changes in either group (*P* > 0.05) (Figure 3).

### Training and Training Quantification

Compared to the lead-in period, there was a main effect of time (*P* < 0.05) for weekly training volume, resulting from a non-significant increase in both groups (HEAT: +13 ± 7%; CON: +11 ± 9%, *P* > 0.05). Also, the weekly training volume above 80% of maximum heart rate increased for HEAT from 102 ± 21 to 157 ± 27 min/week and for CON from 102 ± 18 to 122 ± 19 min/week (both *P* < 0.05 from pre to post, but not significantly different across groups).

### Hematological Parameters

Main effects of time were detected for both BV and PV (both *P* < 0.05, but no time × intervention interaction). For BV with a 5.7 ± 1.5% increase from pre to post in the HEAT group (*P* < 0.01) and a 3.2 ± 1.6% increase for CON (*P* < 0.05) with no significant differences between groups (*P >* 0.05).

From PRE to POST, PV increased by 6.5 ± 2.2% in the HEAT group (*P* < 0.01) and there was a 4.5 ± 2.4% increase in CON (*P* < 0.05), with the overall response not different across groups. As previously mentioned, we refer to the accompanying paper (Oberholzer et al., 2019) for detailed description of the hematological parameters and involved mechanisms.

## Discussion

The prolonged HA period employed in present study was associated with significant sudomotor adaptions (with the reduction in sweat [Na^+^] leveling off after 2–3 weeks of training in the heat), improved exercise endurance in hot environmental settings (i.e., increased TTE at fixed submaximal workload in 40°C), plasma volume expansion and an elevation of total blood volume. However, when transfer effects to endurance performances in cool conditions were tested post HA (in settings with low environmental heat load; i.e., below 15°C), the heat-training group did not increase peak aerobic power, improve submaximal exercise efficiency or VO_2max_, and TT performance effects were similar compared to the matched control group. We refer to the accompanying paper (Oberholzer et al., 2019) for detailed discussion of potential benefits and mechanisms involved in the hematological response observed for the HEAT group, but from the present measures of performance and aerobic power in a population of endurance-trained cyclists, it appears that the potential physiological advantage of a slightly increased Hb-mass was outweighed by the concurrent hemodilution.

To characterize HA status and the gradual heat adaptation, we measured sweat [Na^+^], resting and exercise T_core_ as well as HR responses at the beginning and end of every week of training, and hematological responses were measured at the start and end of the study. End exercise T_core_ and HR decreased during the intervention period, despite an increase in ambient temperature, and PV expanded by ∼7%, as expected following HA (Périard et al., 2016). Further, in the subgroup of six participants who were tested at day 1, 14, and 28 under standardized ∼40°C conditions, TTE was increased from 38.7 min to 64.3 min. For all subject, the total sweat rate or sweat Na^+^ measured during the TT in cool conditions did not increase (from pre to post). This apparent lack of adaptation may be explained by testing in compensable conditions where sweating is dictated by the evaporative requirements (Ravanelli et al., 2018) and due to rapid alterations in the onset and decay of sweat sodium Na^+^ (Williams et al., 1967; Armstrong and Maresh, 1991). Taken together with the lowered resting rectal temperature, these measures provide strong evidence that the participants were successfully acclimated following the prolonged heat training period.

In terms of exercise performance as well as the muscular and cardiovascular adaptations required to improve performance, the present findings are in contrast with previous investigations reporting beneficial effects of heat training for aerobic performance in cool or temperate conditions (Sawka et al., 1985; Buchheit et al., 2011; Neal et al., 2016a; Rendell et al., 2017). One explanation for this discrepancy could be the lack of a control group in these studies or failure to control for training quality in the lead-in phase as well as the during the intervention period. One study (Sawka et al., 1985) showed an increase in both VO_2max_ and iPPO after nine consecutive days of HA (2 h per day at 49°C, 20% RH), while others report improved TT, iPPO or intermittent exercise performance (Buchheit et al., 2011; Neal et al., 2016a; Rendell et al., 2017). If the present study had not included a control group, our findings would be in line with some of these observations, as both the HEAT and CON group improved their TT performance, likely resulting from improved fractional VO_2max_ utilization (Bassett and Howley, 2000), as cycling efficiency and VO_2max_ remained unaffected. Supporting our findings, but with a shorter intervention period, Karlsen et al. (2015b) demonstrated that compared to a matched control group, there was no difference in TT performance, VO_2max_ and iPPO in trained cyclists, after 2 weeks of a heat training camp in hot dry environment (all training in heat group conducted outdoors in Qatar). Collectively, these findings suggest that many of the previous observations suggesting that a period with training in hot conditions improves exercise performance in temperate conditions relates to the physical training or additional training load, rather than the environmental heat stress *per se*.

Supporting this notion, previous studies reporting beneficial effects of heat training for subsequent performance in temperate conditions employed untrained individuals (Nadel et al., 1974; Shvartz et al., 1977; Sawka et al., 1983, 1985; King et al., 1985; Young et al., 1985; Takeno et al., 2001). Of particular relevance, many of these studies reported improved exercise economy following heat training (Shvartz et al., 1977; Sawka et al., 1983; King et al., 1985; Young et al., 1985). Improvements in exercise economy are likely explained by alterations in skeletal muscle fiber type composition and enhanced muscular strength (Corbett et al., 2014). In the present study, which employed a sub-elite cycling population, exercise economy was unchanged by 6 weeks of training in either the control or heat training group. Likewise, Keiser et al. (2015) observed no improvements in either TT performance or VO_2max_ in well trained cyclist in a laboratory-based acclimation study with a cross-over design. This finding further suggests that previous improvements in exercise performance with heat training had more to do with training untrained participants, rather than the direct effect of environmental heat stress.

One exception to the above studies (Lorenzo et al., 2010), reported increases in both TT performance and VO_2max_, compared to a control group, using a sample of endurance trained participants. Both groups performed their usual training outdoors in cool temperate conditions, while adding HA or CON training at low intensity (50% VO_2max_) in the laboratory; however, additional training was not controlled for in either HEAT or CON. In contrast to the study by Lorenzo et al. (2010), both Keiser et al. (2015) with laboratory-based acclimation and Karlsen et al. (2015b) with natural acclimation (all training in outdoor hot environment) report no superior effect of training in the heat compared to control and the overall effect may depend on the “quality” of the participant habitual training (e.g., if the regularly include high intensity training). Since elite endurance athletes in general will optimize and include high quality, and specifically intense training in preparation for competitions, we find it relevant to ensure that high intensity training is included both in the lead-in phase and during the intervention, as it will clearly influence the potential to develop or maintain VO_2max_ in endurance trained individuals (Gormley et al., 2008). How the overall training impact or load (considering both volume and intensity) is quantified and subsequently matched across groups (in studies where superimposed environmental heat stress elevates HR for a given power output) may always be a matter of debate. In the present study we secured that both groups maintained high-intensity intervals in cool settings and although there was an increased weekly time with HR above 80% of maximum compared to the lead-in period, there was no significant differences across groups; neither for total training volume nor time with HR above 80% of maximum during the lead-in period or the intervention phase of the study. Also, HEAT graduate improved heat tolerance and raised cool TT power output during the post-testing indicating that overload (accumulated fatigue) was not limiting their performance or physiological adaptation to training.

Considering that systemic oxygen delivery and hence VO_2max_ depends on both cardiac output and arterial oxygen content (Ekblom, 1986; Bassett and Howley, 2000), it is likely that beneficial effects of an increased blood volume on cardiac filling and cardiac output may be outweighed by lower arterial oxygen content per liter blood induced by the lower [Hb] associated with plasma volume expansion. It should also be considered that the total training volume and weekly volume with HR above 80% of maximum HR increased for both groups during the intervention period. Further, a perfect match between groups (when aiming at maintaining similar volume, intensity and still optimized training) is an issue in studies with superimposed environmental stress, or as intended in the present study, with part of the training substituted by training in the heat. Thus, training quality is a multifaceted matter that may not be adequately quantified by the total volume and/or relative HR intensity. Some participants in the present study indicated that the physiological strain associated with the intervention compromised their ability to uphold habitual high-intensity interval training procedures, and it is well-established that training intensity is imperative toward development and maintenance of VO_2max_ in endurance trained individuals (Gormley et al., 2008). However, considering that HEAT by both measures of training quality (total volume and HR above 80% of maximum) was exposed to similar training load as CON and that they in fact improved TT performance, it is unlikely to be the cause for the unchanged peak power or effects on VO_2max_.

McCleave et al. (2017) reported improvements in 3 km TT, but it should be mentioned that their running TT performance was identical in the heat training and control group in the tests conducted immediately following the 3 week intervention, but superior in the heat group following additional 3 weeks of return to normal training practice. Potentially, timing of the follow-up testing may be important and performance could be optimal in the post acclimatization period, when PV returns toward normal. However, that relies on the premise that total red blood cell mass remain elevated (i.e., red blood cells follows expected life time of ∼120 days; see Kurata et al., 1993) and the normalized PV results in advantageous hemoglobin up-concentration (Convertino et al., 1980). Additionally, heat training purportedly improves exercise performance through both thermal and non-thermal adaptations (Corbett et al., 2014). Performance testing in the present study was conducted in ∼10°C wet bulb globe temperature, and therefore likely did not meet the threshold required to impose a thermal limitation (Junge et al., 2016). However, exercise in environmental conditions on the warmer side could meet the threshold, or rather range, of thermal conditions where improved thermoregulatory capacity induced by HA would be ergogenic. Also, the performance tests applied in the present study (incremental peak power test and the 15 km TT including the preload period) had duration of less than 1 h and potential effects of initiating exercise with increased PV and higher total body water during prolonged physical activities (ultra-sports) cannot be excluded.

## Conclusion

Overall, we conclude that training in the heat was not superior compared to normal (control) training for improving aerobic power or TT performance in cool conditions. However, during a competitive season athletes may be exposed to varying environmental conditions, and although HEAT was not superior compared to CON for improving endurance in cool settings, it is noteworthy that the replacement of a substantial part of overall training volume with heat training did not compromise the effect of training toward temperate exercise performance. Implementation of heat training could therefore be advantageous as part of an integrated pre-season training preparation but the timing of a specific heat training camp may depend the specific competition schedule, considering that the benefit from HA may decay in term of benefitting performance in the heat, whereas for performance in cool settings, a period with return to training without superimposed heat stress may be beneficial.

## Data Availability Statement

All datasets generated for this study are included in the article/supplementary material. Some data can be found in the accompanying article (doi: 10.3389/fphys.2019.01379).

## Ethics Statement

The studies involving human participants were reviewed and approved by the ethics committee of the Capital Region of Denmark (protocol: H-17036662). The patients/participants provided their written informed consent to participate in this study.

## Author Contributions

All authors completed the experimental study [testing and analyses (physiological and statistics)], and drafted the proof of manuscript. CM, NJ, CL, and LN contributed to the design, ethical approval, and development of test/study protocol. LN and CL managed the project.

## Conflict of Interest

The authors declare that the research was conducted in the absence of any commercial or financial relationships that could be construed as a potential conflict of interest.
